# Paternal and Maternal Influences on Differences in Birth Weight between Europeans and Indians Born in the UK

**DOI:** 10.1371/journal.pone.0061116

**Published:** 2013-05-08

**Authors:** Jonathan C. K. Wells, Georgina Sharp, Philip J. Steer, David A. Leon

**Affiliations:** 1 Childhood Nutrition Research Centre, UCL Institute of Child Health, London, United Kingdom; 2 Royal Cornwall Hospitals NHS Trust, Truro, Cornwall, United Kingdom; 3 Academic Department of Obstetrics and Gynaecology, Imperial College London, Chelsea and Westminster Hospital, London, United Kingdom; 4 Department of Non-communicable Disease Epidemiology Unit, London School of Tropical Medicine and Hygiene, London, United Kingdom; Tehran University of Medical Sciences, Islamic Republic of Iran

## Abstract

**Background:**

Ethnic groups differ significantly in adult physique and birth weight. We aimed to improve understanding of maternal versus paternal contributions to ethnic differences in birth weight, by comparing the offspring of same-ethnic versus mixed-ethnic unions amongst Europeans and South Asian Indians in the UK.

**Methodology and principal findings:**

We used data from the UK Office for National Statistics Longitudinal Study (LS) and the Chelsea and Westminster Hospital (CWH), London. In the combined sample at all gestational ages, average birth weight of offspring with two European parents was significantly greater than that of offspring with two Indian parents [Δ = 344 (95% CI 329, 360) g]. Compared to offspring of European mothers, the offspring of Indian mothers had lower birth weight, whether the father was European [Δ = −152 (95% CI −92, −212) g] or Indian [Δ = −254 (95% −315, −192) g]. After adjustment for various confounding factors, average birth weight of offspring with European father and Indian mother was greater than that of offspring with two Indian parents [LS: Δ = 249 (95% CI 143, 354) g; CWH: Δ = 236 (95% CI 62, 411) g]. Average birth weight of offspring with Indian father and European mother was significantly less than that of offspring with two European parents [LS: Δ = −117 (95% CI −207, −26) g; CWH: Δ = −83 (−206, 40) g].

**Conclusions/Significance:**

Birth weight of offspring with mixed-ethnic parentage was intermediate between that of offspring with two European or two Indian parents, demonstrating a paternal as well as a maternal contribution to ethnic differences in fetal growth. This can be interpreted as demonstrating paternal modulation of maternal investment in offspring. We suggest long-term nutritional experience over generations may drive such ethnic differences through parental co-adaptation.

## Introduction

Numerous studies have demonstrated substantial differences in stature, physique and adiposity between Europeans and South Asians. In adulthood, South Asians tend to have lower stature and fat-free mass than Europeans, and to be proportionally more adipose [1]–[3]. South Asians are also lighter at birth than Europeans, even among those born in high-income countries such as the UK [4]. Adult differences may arise in part through patterns of fetal growth, as birth weight is strongly associated with adult height and body composition [5]. For example, detailed comparison of neonates from Southampton (UK) and Pune (India) showed that while the Indian neonates were substantially lighter at birth than the UK neonates (2.7 vs 3.5 kg), they had similar adiposity as assessed from subscapular skinfold thicknesses [6]. Three further studies have replicated these neonatal ethnic differences [7]–[9], though another failed to do so [10]. Most recently, we have established that at 3 months of age among infants born in the UK, those of South Asian ancestry have less fat-free mass than those of European ancestry [11]. Since birth weight variability is considered a key factor contributing to the risk of chronic degenerative diseases in adulthood [12]–[14], ethnic differences in birth weight are a plausible contributing factor to ethnic differences in the prevalence of type 2 diabetes within and across populations [15], [16].

The causes of such ethnic differences in early growth and body composition remain poorly understood. Within populations, variability in adult stature is considered to have a strong genetic component. Twin studies of Europeans have suggested heritability values approaching 90% [17], whilst almost 200 individual alleles have been associated with variability in stature [18]. However, the genetic contribution to stature may have been over-estimated, as twin studies are unable to differentiate epigenetic from genetic effects [19]. Furthermore, whether genetic factors that contribute to variability within populations also account for variability between populations has been questioned [20].

A recent multi-centre study in populations of high socio-economic status showed negligible ethnic variability in growth rate during fetal life and infancy [21]. Despite such similar potential, actual size at birth differs markedly across ethnic groups [22], one likely reason being that ethnicity is strongly associated with socio-economic status and living conditions.

Environmental factors are therefore an alternative contributing factor to ethnic differences in early growth and subsequent physique. Such effects may be exogenous (eg maternal diet, tobacco or alcohol use, or disease load) or endogenous (eg maternal body size or metabolism). For some outcomes such as childhood intelligence quotient, ethnic differences disappear if adjusted for indices of poverty [23], however the situation for birth weight is less clear [24].

Parental contributions to fetal growth are complex, in that the mother provides the uterine environment, while both parents contribute to the fetal genome. One of the first experiments to attempt to disentangle parental genetic from non-genetic contributions to variability in size at birth involved the crossbreeding of Shire horses and Shetland ponies, two strains differing substantially in adult body size. Mating large Shire females with small Shetland males produced offspring larger than those from pure Shetland crosses, but smaller than those from Shire crosses, while crossing Shetland females with Shire males produced offspring no heavier than those of pure Shetland crosses [25]. The authors of this pioneering study assumed the offspring of the mixed breeding would be genetically intermediate, irrespective of which way the cross was made, and considered maternal size to be the primary experimental variable. This study demonstrated a powerful maternal influence on fetal growth [25], and has been widely interpreted as indicating that paternal genetic effects on offspring growth are negligible. Although several subsequent cross-breeding experiments showed that large males induced larger birth weights from small females than did small males [26]–[28], the notion that maternal effects dominate fetal growth has persisted.

Early studies of birth weight variability in humans likewise highlighted the importance of maternal influence on offspring growth. A study of Japanese families found that the birth weight correlation between half-siblings with the same mother (r = 0.58) was substantially greater than that between half-siblings with the same father (r = 0.10) [29]. Similarly, the correlation in birth weight between consecutive siblings (r = 0.52) was greater than that between siblings with one intervening sibling (r = 0.42) or with two intervening siblings (r = 0.36), emphasizing the contribution of maternal non-genetic factors to birth weight variability [29].

Building on the first study of birth weight correlations across generations [30], Ounsted and colleagues compared trans-generational associations in birth weight between families characterized by large or small babies, and concluded that paternal effects were negligible when maternal constraint is severe [31]. These authors proposed that *in utero* growth constraint is transmitted in a non-Mendelian fashion through the maternal line, a finding that has received some support from animal studies [32], [33]. An alternative source of evidence emphasizing the importance of maternal phenotype for human fetal growth is provided by the study of human ovum donation, which show associations of recipient weight, but not donor weight, with offspring birth weight [34].

Subsequent studies, however, have demonstrated independent associations of both parents' birth weights with that of the offspring, indicating moderation of maternal constraint by paternal phenotype. For example, in a study of ∼70,000 father-mother-child trios from Norway, birth weight correlations were 0.226 for mother-child and 0.126 for father-child [35]. A comparison across 3 generations likewise showed a grandparental contrast, with the correlation for grandfathers (r = 0.096) again weaker than the equivalent one for grandmothers (r = 0.125) [36]. However, other studies provide conflicting evidence regarding the relative effect on offspring birth weight of each additional unit of maternal versus paternal birth weight [37], [38].

Our understanding of the contributions of the two parents to fetal growth variability therefore remains incomplete, with both inconsistent evidence and contradictory interpretations. Furthermore, the majority of published human studies have explored the parental contributions to offspring birth weight within populations.

To improve understanding of the difference in birth weight between Europeans and south Asians in the UK [22], we therefore studied the offspring of couples of common or mixed ethnicity. For the south Asian population, our analysis was restricted to those of Indian ethnicity to generate a more homogeneous contrast. We tested the hypothesis that the offspring from inter-ethnic unions would differ in birth weight from those of same-ethnic unions, and that the birth weights of offspring of inter-ethnic unions would be further affected by the ethnicity of the mother and father.

## Methods

Taking advantage of the increasing prevalence in the UK of partnerships between people of Indian and European ancestry, we examined data obtained from the Office for National Statistics Longitudinal Study (LS) or routinely collected from the Chelsea and Westminster Hospital (CWH), West London, where parentage could be classified either as European-European, Indian-Indian, or inter-ethnic unions. Subjects were neonates whose parents described themselves as Indian, or white (referred to here as European), and the subject's inferred ethnicity was the main exposure. Individuals were excluded if either parent described themselves as having mixed ethnicity. Stillbirths and multiple pregnancies were also excluded.

All data were analyzed anonymously. Written consent for the use of the CWH data for research was not taken because at the time the data were collected, it was not intended to use it for research. Subsequently, use of the CWH data for these analyses was approved by Riverside Research Ethics Committee. Ethical approval for the LS was originally given by the Patient Information Advisory Group (replaced by the National Information Governance Board for Health and Social Care in 2008). Our analysis of LS data, using non-identifiable information, did not require ethical approval. The Office for National Statistics board cleared our analyses for publication.

### Longitudinal study

The Longitudinal Study (LS) links data from household census returns with routinely recorded vital events data for a quasi-random, one percent sample of the population of England and Wales [39]. For the purposes of our analysis, we identified subjects whose mother and father could be identified as either white European or Indian (as self-described in the 1991 Census). These were either new members of the LS sample born between 1975 and 2000, or babies born in the same period whose mothers were LS sample members. Data on a range of potential socio-demographic confounders were also obtained from the 1991 Census, including housing tenure (whether residence was owner occupied or not), mother's education beyond age 18 and Registrar General's occupational social class of the mother (6 ordered categories from Professional to Unskilled non-manual). Father's occupational social class was obtained from the subject's birth registration, along with the number of previous live births to the mother within marriage. The latter was used as a proxy for parity, as information on number of live births outside of marriage is not collected at birth registration. As the European women were more likely to be unmarried than the Indian women, parity is likely to be an underestimate in the European mothers. Parity data were only available if parents were married at birth registration, so those with unmarried parents (7.1 percent) were not included in the statistical models. People with missing birth weight data or birth weight less than 500 g (likely to be an error) were excluded (<5% of the sample). Gestational age was not available in this dataset.

### Chelsea and westminster hospital database

The Chelsea and Westminster Hospital (CWH) in West London is part of the National Health Service and serves a relatively affluent population with high ethnic diversity. Its high quality maternity database is frequently used for research purposes. Data are routinely collected and entered into the database, mainly by midwives. At antenatal booking, information on the self-described ethnicity of mother and partner has been collected, although mothers were not asked directly about the paternity of their child. Mother's height and weight were collected at booking, based on self-reports or measured if women did not know. Duration of gestation was assessed by a clinician, using his/her best estimate based on date of last menstrual period or ultrasound scan, whichever seemed more reliable. Parity (defined here as total number of previous live births, still births and neonatal deaths) was obtained from obstetric records. For these analyses we included all live singleton deliveries at the hospital between April 1998 and January 2004, where the mother and father were either Indian or white European. An area-level indicator of socio-economic position was obtained by linking the mothers' home postcode at the time of delivery to the corresponding electoral ward Index of Multiple Deprivation score. Parents' marital status was not entered into regression models because data were missing for 8.1% of subjects.

### Description of confounding variables

The following categories were used in the LS data: maternal age: <25, 25–29, 30–34, 35+ years; number of previous live births within marriage (‘parity’): 0, 1, 2, 3+; father's Registrar General's occupational social class: I, II, III, IV, V, plus a residual category for ‘other or inadequately described’ occupations; mother's occupational social class in 6 categories again; housing tenure: owner-occupied vs. rented/on short lease; maternal education: obtained a post-18 years qualification vs. no such qualification. The following categories were used in the CWH data: mother's age at delivery: <32, 32–34, 34+ years; mother's parity: 0, 1, 2+ (defined here as number of previous live births, still births or neonatal deaths); Index of Multiple Deprivation score in quintiles (quintiles defined within this population); subject's gestational age: 37–38, 39–40, 41 weeks; mother's height to the nearest cm in quintiles: <160, 160–164, 165–166, 167–171, 172+ cm; mother's weight at booking in quintiles: <56, 56–59, 60–63, 64–69, 70+ kg. Subjects without complete data on all covariates were excluded from the multiple regression analysis.

### Statistics

**Figure 1:**
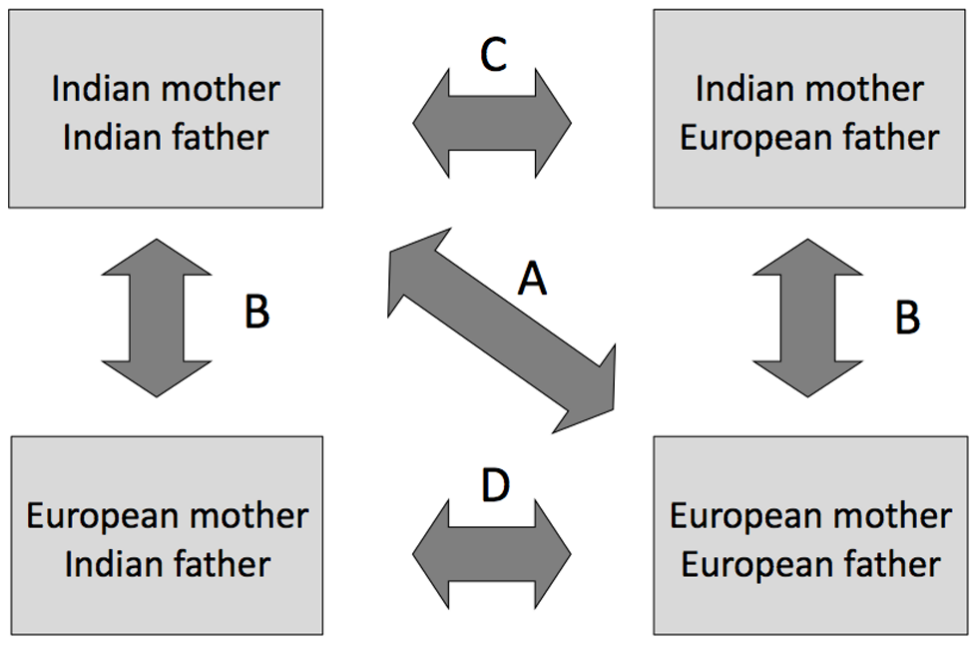
Schematic diagram of the study design, showing the different comparisons undertaken. (A) The crude ethnic difference in birth weight was tested by comparing birth weights of offspring of two Indian versus two European parents. (B) The contribution of Indian mothers to birth weight variability was tested by comparing birth weights of the offspring of Indian versus European mothers, distinguishing whether the father was Indian or European. (C) Whether European fathers promoted the birth weight of offspring relative to Indian fathers was tested by comparing the birth weights of Indian mothers and Indian versus European fathers. (D) Whether Indian fathers constrained the birth weight of offspring relative to European fathers was tested by comparing the birth weights of European mothers and Indian versus European fathers.

We compared birth weight (continuous variable) between each of the four possible categories of union. The study design is illustrated in **Figure 1**, and we refer to the arrows in this figure in the following text. We expected the offspring of two Indian parents to have lower mean birth weight than the offspring of two European parents (arrow A). We assessed the contribution of Indian mothers to birth weight variability by comparing birth weights of the offspring of Indian versus European mothers, distinguishing whether the father was Indian or European (arrow B). We tested whether European fathers promoted birth weight by comparing the mean birth weights of offspring of Indian mothers with Indian versus European fathers (arrow C). We tested whether Indian fathers constrained birth weight by comparing the mean birth weights of offspring of European mothers with Indian versus European fathers (arrow D).

Univariate analyses were performed using one-way analysis of variance (ANOVA) and t-tests for continuous variables, while for categorical variables contingency tables were prepared using Pearson's chi-square test, using Stata 11 (StatCorp: College Station, TX; 2003). Multiple linear regression was performed to adjust for potential confounders (LS: maternal age; parity; paternal social class; housing tenure; maternal education; CWH: maternal age; parity; multiple deprivation score; gestational age; maternal height and weight at booking in quintiles) which were entered as categorical variables. We used robust standard errors to take account of any clustering of birth weight within families.

## Results

**Table 1** presents descriptive data from the LS sample by union type. There were significant differences between groups in maternal and paternal age and maternal parity. Similarly, there were significant differences between groups in type of accommodation, marital status, maternal education, and maternal and paternal social class. **Table 2** presents descriptive data from the CWH sample by union type. There were significant differences between groups in birth weight, gestation length, maternal weight, height and age, and in the Multiple Index of Deprivation score.

**Table 1:** ONS Longitudinal study data: parental characteristics of subjects according to ethnicity of mother and father^a^.

Mother's ethnicity	European		European		Indian		Indian		
Father's ethnicity	European		Indian		European		Indian		
Sample size	121,307		190		136		4,149		
	Mean	SD	Mean	SD	Mean	SD	Mean	SD	P-value^d^
Mother's age (years)	27.7	4.9	29.0	5.2	30.2	5.3	26.8	4.8	<0.001
Father's age (years)	30.3	5.7	32.2	6.6	33.8	7.3	30.0	5.5	<0.001
	%	N	%	N	%	N	%	N	
Mother's parity^b^									P-value^e^
0	41.1	46,212	38.6	64	45.3	58	35.3	1,462	<0.001
1	38.7	43,571	38.0	63	35.2	45	36.3	1,501	
2	14.6	16,461	14.5	24	13.3	17	19.0	786	
3+	5.5	6,214	9.0	15	6.3	8	9.4	389	
Housing tenure									
Owner-occupied	79.0	95,815	83.2	158	80.9	110	88.9	3,690	<0.001
Rented/short-lease	21.0	25,492	16.8	32	19.1	26	11.1	459	
Parents' married status									
Married	92.7	112,458	87.4	166	94.1	128	99.7	4,138	<0.001
Not married	7.3	8,849	12.6	24	5.9	8	0.3	11	
Mother's education									
Post-18 qualifications	17.5	21,173	36.3	69	43.4	59	7.4	306	<0.001
No post-18 qualifications	82.5	100,134	63.7	121	56.6	77	92.6	3,843	
Father's social class									
I & II	33.3	40,354	59.5	113	52.2	71	29.2	1,184	<0.001
III	48.0	56,607	29.2	54	33.1	45	43.2	1,755	
IV & V	17.8	21,032	9.7	18	11.8	16	27.6	1,122	
Other^c^	2.7	3,314	2.6	5	2.9	4	2.1	88	

a Singleton live births (all gestations) 1975 to 2000.

b Parity = number of previous live deliveries. Not available for 8849 (7%) subjects whose parents were not married at registration of their birth.

c ‘Other’ includes occupations inadequately described at the census.

d P-values from one-way ANOVA.

e P-values from Pearson chi-squared tests.

**Table 2:** Chelsea and Westminster Hospital: Gestational age and parental characteristics of subjects according to ethnicity of mother and father^a^.

Mother's ethnicity	European		European		Indian		Indian		
Father's ethnicity	European		Indian		European		Indian		
Sample size	6,101		49		45		69		
	Mean	SD	Mean	SD	Mean	SD	Mean	SD	P-value^b^
Gestation (weeks)	39.7	1.9	40.0	1.2	39.0	2.1	38.1	3.8	<0.001
Mother's height (cm)	165.8	6.8	164.4	7.2	162.4	5.9	159.1	5.8	<0.001
Mother's weight (kg)	63.8	10.3	62.0	8.5	59.5	10.0	58.1	9.5	<0.001
Mother's age (years)	32.8	4.7	34.4	4.2	33.7	4.0	31.3	4.9	0.003
	%	N	%	N	%	N	%	N	P-value^c^
Mother's parity									
0	58.3	3,359	58.7	27	68.3	28	55.2	37	0.321
1	30.4	1,750	30.4	14	29.3	12	38.8	26	
2+	11.4	658	10.9	5	2.4	1	6.0	4	
Fifths of MDI^d^									
I (Least deprived)	19.8	1,135	19.2	9	7.1	3	33.4	21	<0.001
2	21.1	1,212	8.5	4	16.7	7	12.9	8	
3	18.8	1,077	44.7	21	21.4	9	12.9	8	
4	19.5	1,121	6.4	3	26.2	11	14.5	9	
5 (Most deprived)	20.9	1,197	21.3	10	28.6	12	25.8	16	

a Singleton live births (all gestations) 1998–2004.

b P-value from one-way ANOVA.

c P-value from Pearson chi-squared test.

d MDI – Multiple Deprivation Index.

Missing data in sample: height n = 67; weight n = 69; age n = 14; parity n = 343; MDI n = 371.

**Table 3** summarizes the sample sizes and average birth weight by each union type for the LS, CWH and combined samples, unadjusted for confounders. Birth weight differed between the groups (P<0.0001). In the combined sample, average birth weight of offspring with two European parents was significantly greater than that of offspring with two Indian parents [Δ = 344 (95% CI 329, 360) g]. Compared to offspring of European mothers, the offspring of Indian mothers had significantly lower birth weight, whether the fathers in each group were Indian [Δ = −254 (95% CI −315, −192) g] or European [Δ = −152 (95% CI −212, −92) g]. Offspring of the mixed unions had intermediate values. There was a small asymmetry, not statistically significant, according to which parent in the mixed union was Indian, with a lower birth weight if the mother was Indian compared to if the father was Indian [Δ = −61 g (95% CI −160, +37) g].

**Table 3:** Birth weight by parental ethnicity^a^.

	Father Indian			Father European			
	N	Mean (g)	SD (g)	N	Mean (g)	SD (g)	P-value^b^
ONS Longitudinal Survey (LS)							
Mother Indian	4,149	3,044	500	136	3,240	512	<0.001
Mother European	190	3,268	498	121,307	3,383	530	
Chelsea and Westminster Hospital (CWH)							
Mother Indian	69	2,917	779	45	3,216	610	<0.001
Mother European	49	3,402	345	6101	3,451	529	
Combined LS and CWH samples							
Mother Indian	4,218	3,042	506	181	3,234	538	<0.001
Mother European	239	3,295	473	127,408	3,386	530	

a Singleton births, all gestations.

b P-value refers to ANOVA for differences between groups.

The specific impact of paternal ethnicity on birth weight is demonstrated for the LS and CWH samples in **Tables 4** and **5** respectively, taking account of the full range of confounding factors available in each dataset. First, unions between European fathers and Indian mothers, relative to Indian unions, resulted in heavier birth weight in both the LS and CWH studies. Thus, the association of Indian maternal ethnicity with birth weight varies according to paternal ethnicity. Second, both studies also showed that unions between Indian fathers and European mothers, relative to European unions, resulted in lower birth weight, although the association only achieved significance in the LS sample. In the CWH study, the reduction in birth weight was evident after adjustment for offspring sex, gestational age, socio-economic status and maternal age, parity, height and weight. This shows that Indian paternity is associated with lower birth weight compared to European paternity, independent of maternal physical characteristics.

**Table 4:** ONS Longitudinal study: Effect of father's ethnicity on birth weight adjusted for selected covariates^a^.

	Mother Indian				Mother European			
	Father Indian		Father European		Father European		Father Indian	
N	2,892		115		95,578		128	
	Difference (g)	95% CI	Difference (g)	95% CI	Difference (g)	95% CI	Difference (g)	95% CI
Model 1	0	[ref]	253	147, 359	0	[ref]	−91	−206, −21
Model 2	0	[ref]	249	143, 354	0	[ref]	−117	−207, −26

a Singleton live births, all gestations.

Model 1: adjusted for sex.

Model 2: adjusted for sex; Mother's age in 4 categories (<25, 25–29, 30–34, ≥35years); Number of previous live births within marriage in 4 categories (0, 1, 2, ≥3); Father's Registrar General's occupational social class in 6 categories (I, II, III, IV, V, plus a residual category of ‘Other or inadequately described’) and mother's occupational social class (in 6 categories), housing tenure (owner-occupied vs. rented/on short lease), and education beyond age 18 (obtained a post-18 years qualification vs. no such qualification).

**Table 5:** Chelsea and Westminster Hospital: Effect of father's ethnicity on birth weight adjusted for selected covariates^a^.

	Mother Indian				Mother European			
	Father Indian		Father European		Father European		Father Indian	
N	60		38		5,369		42	
	Difference (g)	95% CI	Difference (g)	95% CI	Difference (g)	95% CI	Difference (g)	95% CI
Model 1	0	[ref]	313	103, 524	0	[ref]	−51	−209, 105
Model 2	0	[ref]	290	109, 473	0	[ref]	−61	−198, 74

a Singleton live births, all gestations.

Model 1: adjusted for sex.

Model 2: adjusted for sex; Mother's age at delivery in 3 categories (<32, 32–34, ≥34 years); Mother's parity in 3 categories (0, 1, 2+ previous live births, still births or neonatal deaths); Index of Multiple Deprivation score (at electoral ward level) in quintiles; Subject's gestational age in 3 categories (<39, 39–40, 41+ weeks); Mother's height in 5 categories <160, 160–164, 165–166, 167–171, 172+); Mother's weight at booking in 5 categories <56, 56–59, 60–63, 64–69, 70+ kg).

## Discussion

Our results demonstrate, as expected on the basis of previous research [6], [22], that the offspring of Indian unions are substantially smaller at birth than the offspring of European unions. Going beyond this ethnic comparison, our mixed-ethnic union data offer the possibility to improve understanding of the relative maternal and paternal contributions to this overall ethnic difference in birth weight.

The lower birth weight of offspring of Indian compared to European mothers, whether the father was Indian or European, indicates that Indian mothers contribute substantially to the lower birth weights of Indian neonates. However, since Indian mothers produced a larger baby if the father was European rather than Indian, the magnitude of Indian maternal constraint appears not to be fixed, but to be moderated by paternal effects. In the CWH analyses, these effects were seen even after adjustment for maternal anthropometry, indicating that they are not attributable to differences in maternal size alone. Since European mothers produced a smaller baby if the father was Indian versus European, Indian fathers appear to exert an independent restraining effect on fetal growth, suggesting that they also contribute to the smaller average birth weight of Indian compared to European offspring.

**Figure 2:**
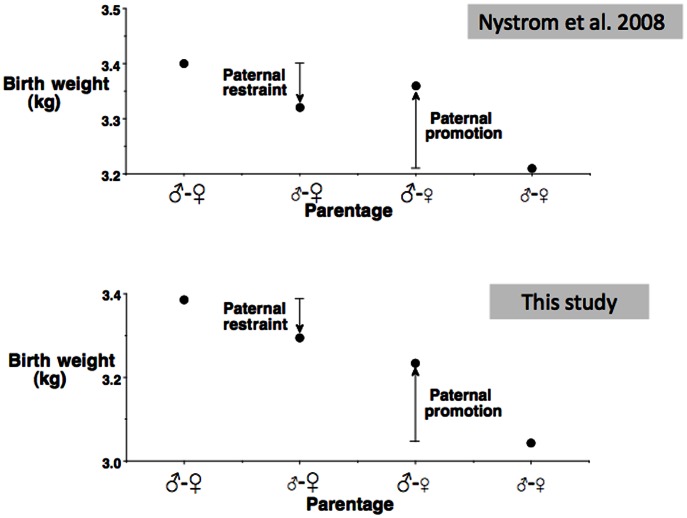
Summary of two studies comparing the birth weight of offspring of unions between different human ethnic groups. The study of Nystrom et al. [40] compared European and Asian populations in the US, whereas our study compared European and Indian populations in the UK. The two parents are denoted by ♂ and ♀ symbols, with large font indicating the European population and small font indicating the Asian or Indian population. Paternal restraint refers to the deficit in birth weight induced by an Asian/Indian father compared to a European father, when exposed to a European mother. Paternal promotion refers to the increment in birth weight induced by a European father compared to an Asian/Indian father, when exposed to an Asian/Indian mother.

We have found only one other study in the literature addressing similar questions [40], although the primary outcome was the risk of caesarian delivery, and birth weight as a continuous variable was not included in any statistical models, or adjusted for maternal anthropometry. Our results are broadly consistent with this earlier study, which showed that, compared to unions between two European parents or two Asian (not further specified) parents, unions between inter-ethnic unions produced offspring with intermediate values. The two studies' findings are compared graphically in **Figure 2**. However, the previous study did not differentiate Asians by region of origin, making this group very heterogeneous. The lower birth weights of our own (South) Asian sample may be lower than the equivalent values from Nystrom and colleagues because we studied Indians, in whom mean birth weight is lower than in Asians in general.

**Figure 3:**
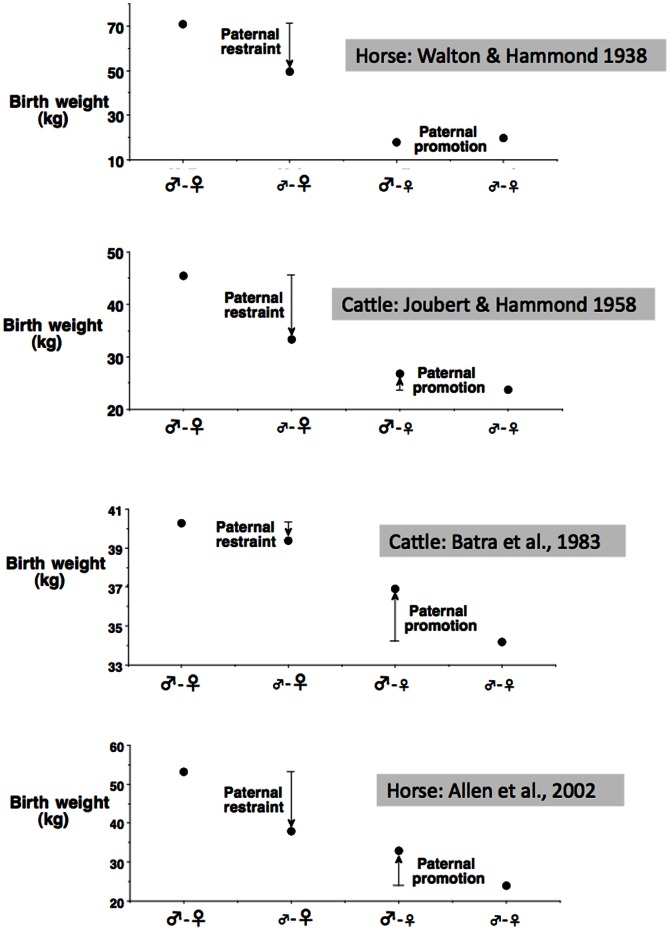
Summary of four studies comparing birth weights following interbreeding between different strains of horses or cattle [25]–[28]. The two parents are denoted by ♂ and ♀ symbols, with large font indicating the strain of larger size and small font indicating the strain of smaller size. Paternal restraint refers to the deficit in birth weight induced by a small father compared to a large father, when exposed to a large mother. Paternal promotion refers to the increment in birth weight induced by a large father compared to a small father, when exposed to a small mother.

More generally, both of these human studies are also consistent with findings from non-human animal studies. Although such work has been widely interpreted as demonstrating the primary influence of maternal phenotype on fetal growth, careful appraisal of the available evidence suggests a more complex scenario. Following the seminal study of Walton and Hammond, a number of further studies were conducted in several animal species, cross-breeding between strains differing substantially in adult body size [26]–[28]. The results are displayed graphically in **Figure 3**. It can be seen that the magnitude of paternal effect differs between the studies. The inability of the small father to take advantage of the larger potential investment of large mothers (paternal restraint) is evident in all studies, though very small in one study of cattle [26]. The capacity of the large father to over-ride the maternal constraint of the small mother (paternal promotion) is minimal in two studies [25], [28] but substantial in two others [26], [27]. It can also be seen that the paternal effects of the large versus small strains are rarely symmetrical.

Our human data are consistent with these general trends, in showing that European fathers generate on average a promoting effect on birth weight of the offspring of Indian mothers, while Indian fathers generate on average a constraining effect on birth weight of the offspring of European mothers. Parallel to the logic laid out by Walton and Hammond [25], if the paternal effects operated purely on a Mendelian basis then birth weight from the two mixed unions would be predicted to be similar, and intermediate between the birth weights of the offspring from each homogeneous union. In the animal studies, each of paternal promotion and paternal restraint is modest, such that offspring of mixed ancestry do not achieve the intermediate birth weights predicted by a Mendelian model. In the two human studies, mixed-ethic birth weights are closer to the expected intermediate values. The greater birth weight deficit attributable to Indian mothers versus Indian fathers, relative to European parents, is consistent with other studies reporting that, within a population, maternal traits generate stronger coefficients on birth weight than do paternal traits [35], [37], though see [38].

However, the paternal effects of our two ethnic groups were also asymmetrical, with the average European ‘paternal promoting’ effect (+240 g) twice the magnitude of the average Indian ‘paternal restraining’ effect (−90 g). This asymmetry is difficult to interpret, as it might indicate a combination of Mendelian genetic effects, parent-of-origin genetic effects, and epigenetic effects reflecting environmental differences. For example, Indian mothers living with European fathers may have experienced different living conditions compared to their counterparts living with Indian fathers. Despite these challenges in interpretation, our data offer support for the proposition that a proportion of ethnic variability in birth weight is due to paternal effects.

Our study has some limitations, notably the small sample sizes of the mixed-ethnic groups, and the inability to adjust fully for parental size and factors such as maternal smoking or pregnancy weight gain. We do not believe paternity uncertainty has adversely influenced our findings, as the frequency of uncertainty must typically be high to cancel out alternative interpretations [37], and if paternity uncertainty was equivalent to incorrect paternal ethnicity assignment, we would expect to have found smaller differences between the parental unions, hence our findings are conservative. Moreover, our results are consistent with both the animal studies comparing populations of differing average size, and with a previous study of inter-ethnic unions between Europeans and Asians [40]. Therefore, the direction of the effects of parental ethnicity demonstrated in our study are likely to be real, although larger sample sizes are required to estimate the magnitude of these effects more precisely. Of particular note, in the CWH sample, adjusting for a number of confounding factors related to maternal size made very little difference to the magnitude of effect.

### Evolutionary interpretation

The different parental contributions to offspring growth *in utero* can usefully be considered from an evolutionary perspective. Trivers [41] proposed that parents and offspring are subject to a conflict of interest over the optimum level of parental investment in the offspring, which in fetal life is supplied physiologically only by the mother. According to this approach, paternal genes in the offspring should promote maternal investment in the offspring, whereas maternal genes in the offspring and mother should constrain investment at a lower level so as to retain resources for future offspring. The theory is widely used to test hypotheses in non-human species, and is supported by substantial experimental research on intra- and inter-brood conflict [42]–[44]. In humans, support for the theory derives from the fact that the commonest birth weight is below that which minimizes neonatal mortality, indicating that the average neonate has received suboptimal maternal investment [45].

Such tension makes possible the evolutionary co-adaptation of paternal demand to maternal supply [46], [47], as recently demonstrated experimentally in birds [48] although the dynamics of such co-adaptation may vary across species. The simplest model of such co-adaptation would assume natural selection of genetic factors affecting fetal growth according to the principles of Mendelian inheritance. On this basis, chronic under-nutrition of the matriline over multiple generations would induce a corresponding reduction in paternal demand for maternal investment.

In a more sophisticated approach, Haig developed Trivers' ideas by focusing on the contribution to fetal growth of imprinted genes, differentially expressed in the offspring according to paternal or maternal origin [49]. Moore and Haig proposed that offspring and maternal genes engage in a tug of war [49], with the offspring manipulating uterine vasculature, placental hormones, and hemodynamics to increase maternal investment, and the mother responding in order to cap it [50]. The large number of imprinted genes expressed in the human placenta [51] is consistent with the conflict hypothesis, however, parent-offspring conflict is not the only theoretical explanation proposed to account for genomic imprinting, and this issue remains debated. Environmentally-induced epigenetic adaptations of paternal demand are another possible mechanism for such effects [52], although human evidence remains scarce [53].

More compelling evidence for the physiological mechanisms of paternal influence on fetal growth come from endocrinological studies. The placenta is able to release hormones (including some moderated by paternal genotype) directly into the maternal bloodstream [50]. Replication of the classic horse study of Walton and Hammond has demonstrated that placental hormones act on maternal receptors [54], and that paternal genes drive growth of the placenta [27]. Each of these processes subjects maternal metabolism to paternal genetic influence [55], [56]. There are some indications that the conceptus can respond within the duration of pregnancy to maternal state, for example developing a larger placenta and thereby raising placental hormone production, in response to maternal characteristics such as anemia [57]. Irrespective of such plasticity, our data suggest that in Indians, the intensity of fetal hormonal output has systematically decreased, such that paternal demand has adapted to lower levels of maternal resources.

An explanation for this lower demand of Indian offspring may lie in the contrasting nutritional histories of European and Indian populations. Recent archaeological evidence indicates that stature in India has decreased substantially over the last 8000 years [58], whereas equivalent declines in European stature are more modest [59]. Over the 4000 year period in which foraging was replaced by early agriculture, male stature in India fell by ∼10 cm, and physique became more gracile [58], [60]. Whilst a degree of decline in stature has been observed in most global regions, and has been attributed to the detrimental dietary effects of adopting crop agriculture [59], [61], the magnitude of the effect in the Indian subcontinent was extreme, and a further ∼10 cm decline occurred between 4000 BP and the start of the 20^th^ century [58], [62]. Two factors may have been important contributors to such drastic declines on the Indian sub-continent. First, regular famines have affected the sub-continent, driven by monsoon disruptions arising from El Nino effects [63] though recently exacerbated by imperial economic policies [64]. Second, perhaps as an adaptation to such energy stress, vegetarianism became widespread after the emergence of Buddhism and Jainism in the 6^th^ century BCE [65].

Under conditions of energy scarcity, larger offspring are predicted to place greater stress on the maternal energy budget and hence threaten both maternal survival, and that of their existing and potential future siblings [66], [67]. Thus, downward secular trends in maternal size and weight could plausibly induce an adaptive response in the level of paternal ‘demand’ for offspring growth, by any of the genetic or epigenetic pathways described above. Our data are therefore consistent with this evolutionary approach, which would suggest that body size has declined in Indian compared to European populations, through genetic and/or epigenetic mechanisms impacting fetal growth.

### Health implications

The notion that adaptations to population energy stress may occur within the developmental niche of maternal physiology, and through the mechanism of parent-offspring dynamics, may shed new light on the way in which ecological pressures induce evolutionary change in our species. Because growth is canalized from late infancy onwards [68], [69], secular trends in stature and lean mass are strongly dependent on growth during fetal life and infancy [70] and are therefore sensitive to maternal nutritional status. Recent studies associating rapid infant weight gain with subsequent chronic disease risk [71], [72] demonstrate the penalties to the offspring if such maternal regulation is diminished by behaviours such as formula-feeding.

Our findings are also relevant to the lack of evidence of birth weight increases in south Asians (Bangladeshis, Pakistanis or Indians) born in the UK, compared to those born in their country of origin [73]. The average 300 g deficit in birth weight of South Asian relative to white British offspring at 40 weeks gestation is doubtless due in part to differences in maternal size, but may also represent the effect of genetic factors as suggested by our analysis. However, any non-genetic effects might still resolve over subsequent generations, as demonstrated in primates over 5 generations [33]. Finally, our findings are relevant to understanding the high risk of diabetes in Indians, which has been linked to their low birth weight [74]. Our findings, in combination with those from migration studies [73], offer little support for the hypothesis that birth weight in Indians will increase rapidly in response to changes in maternal environment, because each parent contributes to the smaller birth weight of Indian neonates, and part of this contribution derives from genetic factors.
